# The Use and Safety of TNF Inhibitors during Pregnancy in Women with Psoriasis: A Review

**DOI:** 10.3390/ijms19051349

**Published:** 2018-05-03

**Authors:** Cæcilie Bachdal Johansen, Espen Jimenez-Solem, Ann Haerskjold, Freja Lærke Sand, Simon Francis Thomsen

**Affiliations:** 1 Department of Clinical Pharmacology, Copenhagen University Hospital Bispebjerg, 2400 Copenhagen NV, Denmark; Espen.Jimenez.Solem@regionh.dk; 2 Department of Dermato-Venereology, Copenhagen University Hospital Bispebjerg, 2400 Copenhagen NV, Denmark; annhaerskjold@gmail.com (A.H.); simon.francis.thomsen.02@regionh.dk (S.F.T.); 3 Danish Cancer Society Research Center, 2100 Copenhagen Ø, Denmark; Frk.Sand@hotmail.com; 4 Department of Biomedical Sciences, University of Copenhagen, 2200 Copenhagen NV, Denmark

**Keywords:** Tumor Necrosis Factor, TNF inhibitors, Tumor Necrosis Factor inhibitors, anti-TNF agents, inflammation, drug safety, psoriasis, pregnancy, neonatal, congenital malformations

## Abstract

Psoriasis is a chronic immune-mediated inflammatory disease affecting women of childbearing potential. Biologic agents, notably Tumor Necrosis Factor inhibitors (TNFi), are the only current non-contraindicated systemic treatment option during pregnancy. TNFi comprised of complete immunoglobulin G (IgG) antibodies antibodies (adalimumab, golimumab, and infliximab) actively cross the placenta from the second trimester and are detectable in the child up to one year postpartum. Data on safety of TNFi are conflicting; however a trend towards drug-specific harm has been reported, with increased risk of congenital malformations and preterm birth. TNFi exposure may alter the immune system of the infant towards hypersensitivity and reduced response to intracellular infections. Confounding by indication should be considered, as chronic inflammatory disease itself may pose a risk of adverse pregnancy outcomes. The quality of the current evidence is very low and no studies specifically address TNFi safety in women with psoriasis. Nonetheless, risks associated with TNFi treatment must be balanced against the as-yet uncertain risk of adverse outcomes in infants born to women with severe psoriasis. We searched PubMed using Medical Subject Headings (MeSH) terms and identified relevant studies and guidelines. Herein, we present the current knowledge of the use and safety of TNFi during pregnancy in women with psoriasis.

## 1. Introduction

Psoriasis is a chronic immune-mediated inflammatory disease that affects 2–4% of the world’s population [1]; approximately 50% are women and nearly 75% of cases are present before the age of 40 [2,3]. Thus, the majority of women with psoriasis are of childbearing potential. 23% of women with psoriasis experience a worsening of their psoriasis with a potential need for treatment [4] and psoriasis itself has been associated with adverse pregnancy outcomes [5]. However, management of psoriasis in pregnant woman is challenging and should balance potential effects and maternal and fetal risks.

Since the late 1990s, TNF-inhibitors (TNFi), in particular adalimumab, etanercept and infliximab, have become a cornerstone in the treatment of psoriasis. These three TNFi accounted for about 25% of the use of psoriasis treatment in a 2013 survey performed in the US [6]. Consequently, women with psoriasis will at some point become or wish to become pregnant during treatment with TNFi.

Knowledge of safety of TNFi during pregnancy is limited but expanding [7]. It is ethically unsound to include pregnant women in randomized controlled trials investigating drug safety. Thus, safety of drugs during pregnancy often relies on post-marketing register-based and case studies. The current first-line systemic therapies methotrexate, acitretin, and to some extent cyclosporine are all contraindicated for women planning pregnancy, due to their teratogenicity and risk of maternal hypertension, low birth weight, and prematurity [8,9].

The inflammatory process and lifestyle factors in women with psoriasis may pose an individual risk to the pregnant mother and child [10,11] and possibly this risk may be higher if the condition is left untreated.

Therefore it is crucial for doctors to have an up to date knowledge both of the natural course of psoriasis and of treatment with TNFi during pregnancy to assess the risks and benefits in each individual case.

Herein, we present an overview of the current knowledge of the use and safety of TNFi during pregnancy in women with psoriasis.

Research question: Are TNFi safe to use during pregnancy in women with psoriasis?

## 2. The Role of Tumor Necrosis Factor (TNF) in Psoriasis

T helper (Th) cells Th1 and Th17 are predominantly responsible for the inflammatory immune response in psoriasis by inducing a cascade of inflammatory cytokines, notably tumor necrosis factor (TNF) [12,13]. 

TNF is the driving force behind initiation and maintenance of the inflammatory process and psoriatic skin is infiltrated with a myriad of immune cells showing elevated expression of TNF including epidermal keratinocytes, intra-epidermal Langerhans cells, papillary dermal macrophages, and upper dermal blood vessels [12,14,15]. Furthermore, patients with polymorphisms in TNF-related genes, e.g., TNFAIP3, respond better to therapy with TNFi [13].

## 3. Mechanism of Action of TNF-Inhibitors in Psoriasis

In 1999, the first TNFi, infliximab, was authorized by the European Medicines Agency for treatment of psoriasis and psoriasis arthritis among other inflammatory diseases. Since then etanercept (2000), adalimumab (2003), golimumab, and certolizumab pegol (2009) were authorized. TNFi works by antagonizing and thereby neutralizing the activity of transmembrane and soluble TNF, thus preventing its binding to the two different TNF-receptors Tumor Necrosis Factor receptor (TNFR)1 and TNFR2. TNFi often works in a dose-dependent manner, blocking the pro-inflammatory activities of TNF [16,17]. The clinical effects of TNFi are gradual disease control and improvement in Psoriasis Area and Severity Index (PASI) score [16]. 

## 4. Psoriasis and Pregnancy

Psoriasis fluctuates in pregnancy and 55% of women with psoriasis reported an improvement in skin symptoms, 23% reported worsening, and 21% reported no change [4]. A substantial proportion of patients with psoriasis develop obesity, hypertension, diabetes mellitus, depression, and other chronic inflammatory disease such as inflammatory bowel disease and rheumatoid arthritis all of which have been associated with adverse pregnancy and birth outcomes.

Psoriasis may pose a risk of adverse pregnancy outcomes. Until recently, there has been no clear evidence found of increased adverse pregnancy and birth outcome in women with psoriasis [10]. Unfortunately, these studies did not stratify for drug use, thus the effect of medication on pregnancy outcomes were not considered [7,10]. 

Active disease and the underlying immunopathology could contribute to overall adverse pregnancy outcomes in women with psoriasis. Yang et al. [18] found that mothers with severe psoriasis (who had received photochemotherapy or systemic therapy within two years of indexed delivery) had a 1.40-fold increased risk of giving birth to low birth weight infants. These findings are supported by a newly published Scandinavian population-based cohort study [5]. They found that women with psoriasis had an increased risk of gestational diabetes, gestational hypertension, preeclampsia and elective and emergency cesarean section. The risks were even higher in women with severe psoriasis (receiving systemic therapy with at least one dispensed prescription), who also had an increased risk of pre-term birth and low birth weight. This suggests that an increased systemic immune activity in psoriasis could be the cause.

### Immunological Shifting During Pregnancy 

The pathogenesis underlying psoriasis’ effect on pregnancy is poorly studied. Increased activity of Th17 and to some extent Th1 cells plays a critical role in the immune pathogenesis of psoriasis [13]. Changes in Th1, Th17, and regulatory T (Treg) cells govern the successful pregnancy, with Treg yielding an immunosuppressive role, inducing tolerance to the pregnancy, especially during the first trimester. An overweight of Th17 cells in the Th17/Treg ratio leads to poorer pregnancy outcomes, e.g., preterm birth, preeclampsia, and unexplained recurrent pregnancy loss, in women with autoimmune disease, such as rheumatoid arthritis, multiple sclerosis, and systemic lupus erythematosus [19].

## 5. Transplacental Transport of TNF-Inhibitors During Pregnancy

Maternally-acquired antibodies are critical to provide immunity to the newborn and protect it against infections during the first months of life before its own immune system matures [20].

Complete immunoglobulin G (IgG) antibodies—both maternal and therapeutic—cross placenta via active transport facilitated by the neonatal fragment crystallizable (Fc) receptor on the placenta. This occurs after the twenty-second week of gestation and throughout the pregnancy, where fetal levels surpass maternal levels of IgG [21,22]. IgG1 is the most effectively transported of the four subclasses of IgG (G1–G4) [21].

This is relevant to adalimumab, golimumab, and infliximab which are complete IgG1 anti-TNF antibodies, and to a smaller extent etanercept that only contains the IgG1 Fcportion. A summary of indications and transplacental transport of the different TNFi is listed in Table 1.

### 5.1. Adalimumab, Infliximab, and Golimumab

All three of these TNFi are complete IgG1 antibodies, thus transplacental transport is expected. An international multicenter prospective cohort study of 80 pregnant women with inflammatory bowel disease (IBD) who either received adalimumab or infliximab, found an inverse correlation between the time from last drug exposure during pregnancy and concentration in cord blood. Mean time for drug clearance in the infants was 4 months for adalimumab and 7.3 months for infliximab. The latter was detectable up to 12 months of age [23].

### 5.2. Etanercept

Etanercept is comprised of the Fc domain of human IgG1 fused with the extracellular ligand binding domain of human tumor necrosis factor receptor-2. Transplacental transport via the neonatal Fc receptor would theoretically be plausible. However, a case report found an etanercept concentration ratio between maternal blood and umbilical cord blood of 14:1 at delivery, in a woman with ankylosing spondylitis receiving etanercept 25 mg subcutaneously once weekly during the second and third trimester [24]. This supports low transplacental transport in concordance with a previous case report [25].

### 5.3. Certolizumab Pegol

In contrast to the complete IgG1 anti-TNF antibodies, infliximab, golimumab, and adalimumab, certolizumab pegol differs structurally as it is a humanized PEG (polyethylene glycol)-ylated antibody Fab’ fragment lacking the IgG1 Fc portion [26]. Without the Fc portion it should, in theory, not be transported actively across placenta by the neonatal Fc receptor, leaving passive diffusion as the only explanatory option for any detectable concentrations in exposed infants. This theory was supported by previous case series [27,28] including a recent case series of women with chronic inflammatory diseases treated with certolizumab pegol during pregnancy. Levels of certolizumab pegol at delivery in the 14 infants ranged from undetectable to one infant with minimal certolizumab pegol levels of 0.042 µg/mL compared with average maternal plasma levels of 24.4 µg/mL, showing an infant/mother plasma ratio of 0.0009 [29].

## 6. Clinical Recommendations

The current TNFi indicated for psoriasis and psoriasis arthritis are adalimumab, infliximab, golimumab, etanercept, and certolizumab pegol, respectively. The British Association of Dermatologists states in their guideline that decision making on treatment during pregnancy should be made on a case by case basis, with no defined gestational cutoff for drug discontinuation. The guideline underlines that live vaccination (e.g., rotavirus and Bacillus Calmette–Guérin (BCG)) should be avoided in infants of mothers taking biologic therapy beyond gestational week 16 [36]. 

Women with IBD are at increased risk of adverse maternal and neonatal outcomes if their disease is active during pregnancy. The European Crohn’s and Colitis Organization recommends in their consensus statement that these women are best treated appropriately and without delay. And if the disease activity allows it, treatment with TNFi should be discontinued around gestational week 24–26 [37].

Consensus across these guidelines is that pregnant women with an inflammatory disease should receive multidisciplinary care involving a team with experience in handling women with active disease during pregnancy and in the postpartum period.

## 7. General Safety of TNF Inhibitors During Pregnancy

Most TNFi safety studies have been conducted in indirect populations, such as women with IBD and rheumatoid arthritis, consequently no studies specifically address TNFi potential risk of harm in pregnant women with psoriasis or the outcomes in their infants [7].

### 7.1. Adverse Maternal Outcomes

Previous safety studies often focus on composite adverse outcomes both regarding the pregnancy itself and the infant, but to a far less extent investigating the potential risks for the mother [32]. Adverse maternal outcomes are defined as adverse obstetrical events during pregnancy e.g., preeclampsia, essential thrombocythemia, gestational diabetes, hypertension, infections, venous thromboembolism, intensive care unit admission, and cesarean section. 

Preeclampsia is considered to be an exacerbation of the normal inflammatory response present in the last trimester of the pregnancy [19,38]. In a retrospective multicenter study in pregnant women with IBD, the rate of preeclampsia was similar between patients exposed to TNFi and non-exposed patients [31]. The role of TNF in the pathogenesis of preeclampsia is not yet fully understood. Nonetheless a correlation between preeclampsia and elevated circulating maternal TNF and umbilical levels of the soluble form of TNF receptor 1 has repeatedly been found [11,38,39], suggesting that treatment with TNFi, theoretically, could protect against preeclampsia. To our knowledge no further studies address adverse maternal outcomes in TNFi-exposed women.

### 7.2. Adverse Pregnancy Outcomes

Adverse pregnancy outcomes have been examined in TNFi-exposed pregnancies and are often defined as spontaneous abortion, elective termination, ectopic pregnancies, intrauterine death, and still birth. A meta-analysis in TNFi-exposed women with IBD found that the rate of elective terminations was 17% compared to 0.02% in the background population. Spontaneous abortions were 12% in the TNFi-exposed pregnancies compared to 20% expected and there were no ectopic pregnancies or still births registered compared to a normal population incidence both of 1% [40]. In contrast, Casanova et al. found that pregnancies where the TNFi drugs (adalimumab, certolizumab, or infliximab) were discontinued within the first trimester, had a significantly higher frequency of spontaneous abortions compared to pregnancies exposed to TNFi throughout all three trimesters [31].

Confounding by indication—the fact that the disease itself and not necessarily the drug indicated for the disease, can cause a given adverse outcome—may be a part of the explanation to why higher rates of abortions are reported in women with chronic inflammatory diseases. Active disease at time of conception has been associated with increased rates of spontaneous abortions in women with IBD [32]. And control of disease activity is the most effective way to improve their pregnancy outcomes [36]. Thus, treatment with TNFi in women with severe active disease may be an acceptable option for these women to achieve a successful pregnancy.

### 7.3. Adverse Neonatal Outcomes

The words fetal and neonatal adverse outcomes are often used interchangeably and cover congenital malformations, preterm birth, low birth weight, small for gestational age, intrauterine growth retardation, respiratory distress syndrome, neonatal infections, admission to neonatal intensive care unit, and death. 

The most recent systematic review found a trend towards drug-specific harm with increased risk of major congenital malformations and preterm birth in infants of women with other chronic inflammatory diseases than psoriasis exposed to TNFi [7]. Supporting these findings, a Scandinavian register-based cohort study found a non-significantly higher risk of congenital malformations among women with IBD treated with TNFi. However, there was no distinct pattern of observed malformations to indicate an underlying mechanism—which would otherwise be expected for a teratogenic drug [30].

In contrast, a 2016 meta-analysis found no increased risk of congenital malformations in TNFi-exposed women with IBD compared with disease-matched controls [32]. Furthermore, the relative risk of congenital malformations in adalimumab-exposed women with rheumatoid arthritis was similar to that of disease matched controls and healthy women [34].

### 7.4. Low Birth Weight and Preterm Birth

Low birth weight is defined as a birth weight less than 2500 g. However, this definition does not take gestational age, sex, race, and clinical features into account [41]. Thus, low birth weight is an umbrella term that also encompasses children born small for gestational age (SGA) and intrauterine growth retarded (IUGR). The distinction is not without relevance as SGA and IUGR have been associated with chronic inflammatory disease of the mother [42,43].

The presence and activity of TNF in both mother and fetus during pregnancy seems to play a leading role in the determination of birth weight. Increased maternal TNF-levels and placental TNF-production have been associated with low birth weight and IUGR [11,39,44].

A prospective observational multicenter cohort study, investigated pregnancy outcomes in women with chronic inflammatory diseases treated with TNFi compared to non-exposed non-disease matched random sampled pregnant women. They found a significantly increased risk of preterm birth and low birth weight in the exposed pregnancies. Unfortunately the control group was not disease-matched—hence the increased risks of adverse neonatal outcomes could either be due to the disease itself (confounding by indication) or a result of TNFi exposure [33]. 

Contradicting these findings, a following meta-analysis found that TNFi treatment did not increase the risk of preterm birth nor low birth weight in children of women with IBD compared with disease-matched controls [32].

### 7.5. Neonatal Infections and Immunological Changes

Due to accelerated active transplacental transport in the second and third trimester, treatment with TNFi during pregnancy is hypothesized to lead to neonatal immunosuppression with increased risk of neonatal infections. A tragic case of neonatal death due to disseminated BCG infection occurred after BCG vaccination in an infant born to a mother treated with infliximab throughout pregnancy [45]. Following, the Center of Disease Control and Prevention recommended that administration of live vaccinations should be avoided in full-term newborns exposed to TNFi, for at least 6 months postpartum. However, they can receive inactive vaccinations as scheduled in the vaccination program [46].

How fetal TNFi exposure potentially may alter the development of their immune system is poorly studied. A clinical immunology study from 2017 examined seven infants exposed to either adalimumab or infliximab throughout pregnancy. They presented more immature B- and Th cells that normalized within the first year of life. A decreased frequency of Tregs in the infants at birth was inversely correlated with maternal peripartum TNFi levels.

Furthermore, a reduced response to mycobacteria was found and four infants developed atopic dermatitis within the first year of life. Esteve-Solé et al. hypothesized that the observed decrease in Tregs could facilitate hypersensitivity leading to atopy, and defects in interleukin 12/interferon-gamma pathways could make TNFi-exposed infants more susceptible to intracellular infections caused by e.g., mycobacteria such as the tuberculosis bacteria BCG [47].

Contrasting these findings, an animal study in the macaque monkey exposed to golimumab throughout pregnancy showed no effect on T and B cells and a normal immune response to antigen challenge in the infants [35].

### 7.6. Long-Term Safety

Long-term safety of in utero exposure to TNFi is another concern with limited evidence despite nearly 20 years on the market [48]. Relevant long-term outcomes could be severe infections, atopy, allergies, and malignancies (especially lymphoid cancers) all indicating possible influence on the developing immune system [49].

With a mean follow-up of 4 years Chaparro et al. [48] found no cases of cancers and no significantly increased risk of severe infections (leading to hospital admission) among exposed children of TNFi-treated mothers with IBD compared to disease matched controls.

To our knowledge, only one case series (data reported to the American Food and Drug Administration) reports two cases of malignancies: leukemia (age 4) and neuroblastoma (less than 1 year old) in children exposed to TNFi in utero [50]. 

## 8. Discussion

Despite the growing numbers of observational studies regarding safety of TNFi in pregnancy, there still exists a critical gap in our knowledge specifically addressing safety in women with psoriasis and long-term outcomes in the exposed children. The available studies indicate the following:

1. No studies investigate adverse pregnancy outcomes in TNFi-exposed women with psoriasis [7] and very few studies address adverse maternal outcomes [5,31,36].
2. The current psoriasis guidelines refers to no gestational cutoff for TNFi discontinuation and emphasize that the decision should be made on a case by case basis [36].
3. Chronic inflammatory diseases (psoriasis, IBD, and rheumatoid arthritis) are associates with adverse pregnancy outcomes [10,19,36,37].
4. TNFi comprising of complete IgG1 antibodies (adalimumab, golimumab, and infliximab) actively cross placenta from the second trimester and are detectable in the child up to one year postpartum [21,22,23].
5. Data on elective terminations and spontaneous abortions are conflicting, however TNFi treatment can yield a successful pregnancy in severe cases of IBD [37].
6. TNFi-exposed infants of women with chronic inflammatory diseases trend towards drug-specific harm with increased risk of major congenital malformations and preterm birth [7,30].
7. TNFi exposure may alter the immune system of the infant towards hypersensitivity and reduced response to intracellular infections [47].
8. Confounding by indication should be considered when studying adverse pregnancy outcomes in TNFi-treatment.
9. The risks associated with TNFi-treatment must be balanced against the as-yet uncertain risks of adverse pregnancy outcomes in infants born to women with severe psoriasis [36,51].

Major congenital malformations can occur due to exposure to teratogenic agents during the organogenesis from gestational week three to eight, whereas functional defects and minor malformations can occur during the fetal period week 9–38 [52]. Adalimumab, golimumab, infliximab, and to a lesser extent etanercept are all actively transported across placenta from gestational week 22, thus it is logical to expect minor malformations from exposure to these drugs, rather than major congenital malformations. It could be suspected that minor malformations and long-term adverse effects such as mental, intellectual or sensory impairments, and lymphoid cancers are being underreported [53]. Nonetheless, we must base our safety assessment on the actual reported congenital malformations, comprising an ethical dilemma if unintended yet systematic underreporting is the case.

### 8.1. Quality of Evidence

Safety assessment of treatment with TNFi during pregnancy is primarily based on observational studies, which inherently have low level of evidence. The current studies are weakened by

- large heterogeneity between studies—limiting generalizability of their findings
- indirectness (studies were performed in women with IBD and rheumatoid arthritis, not explicitly including women with psoriasis)—thus findings may not be applicable to women with psoriasis
- imprecision with wide confidence intervals on adverse outcomes
- often underpowered and some lacking disease-matched controls and multivariate adjustment.

Using Grading of Recommendations Assessment, Development and Evaluation (GRADE) Pottinger et al. [7] classified the quality of evidence of their included studies as ‘very low’ and large heterogeneity prevented a meta-analysis.

### 8.2. Future Challenges

More than a fifth of women with psoriasis experience a worsening of their skin symptoms during pregnancy leaving a potential need for treatment [4]. Low- to moderate-potency topical steroids are the recommended first-line treatment for pregnant women with limited psoriasis [54], however, data on their safety is still limited and inconclusive. A 2010 Cochrane review found an association of very potent topical corticosteroids with low birthweight [55].

First-line systemic therapies methotrexate and acitretin are contraindicated for women planning pregnancy due to their highly teratogenic effects. Cyclosporine has been associated with increased risk of maternal hypertension, low birth weight, and prematurity [8,9].

Newer biologics indicated for psoriasis are:

- Interleukin (IL)-17 antagonists: secukinumab (IgG1 mAb), ixekizumab (IgG4 mAb), and brodalumab (IgG2 mAb)
- IL-12/23 antagonist: ustekinumab (IgG1 mAb)
- IL-23 antagonist: guselkumab (IgG1 mAb).

They are all IgG monoclonal antibodies, thus expected to be transported actively across placenta during the second trimester like the IgG TNFi adalimumab, golimumab, and infliximab. However, their mechanism of action are different from TNFi. Evaluation of newer biologics during pregnancy can be performed through retrospective post marketing non-interventional studies comparing them with TNFi.

A case series described use of TNFi and the IL-12/23 antagonist, ustekinumab, with no adverse pregnancy outcomes or negative effects on the infants [51]. Yet, no controlled human trials have investigated the safety of IL-17, IL-12/23, and IL-23 antagonists during pregnancy, leaving TNFi as the only non-absolute contraindicated biologic treatment option [7]. 

Although psoriasis can flare during pregnancy it rarely becomes an acute life-threatening condition. Thus, dermatologists experience that women with psoriasis often wish to discontinue systemic treatment prior to conception to avoid unnecessary drug exposure of the fetus. This notion is supported by a systematic review on drug survival of biologic treatment in psoriasis, which showed that pregnancy was a cited reason for drug discontinuation [56]. This fact may compromise recruitment of pregnant women with psoriasis in future randomized controlled trials investigating safety of biologics during pregnancy. At present, we base our clinical decision making on very low grade evidence [7]. Furthermore, large cohort studies in women with psoriasis are needed with

- disease-matched and healthy controls
- adjustment for all drug use and confounders such as disease activity and patient demographics
- long-term follow-up of >10 years to uncover potential immunological defects, lymphoid cancers, or mental and intellectual impairments among the exposed children.

Only then, can we make an informed decision on treatment with TNFi during pregnancy based on sound evidence.

## 9. Materials and Methods

We searched PubMed using the following MeSH terms (“psoriasis” OR “psoriasis arthritis” OR “psoriasis arthropathy”) AND (“tnf inhibitors” OR “tnf antagonists” OR “tumor necrosis factor alpha inhibitor”) AND (“pregnancy” OR “newborn” OR “neonatal”). We then identified meta-analysis, systemic reviews, narrative reviews, observational studies, and guidelines and selected relevant English-language publications based on their abstracts. Additionally, we found relevant guidelines and studies from the reference lists of the initially included publications.

## Figures and Tables

**Table 1:** Summary of Tumor Necrosis Factor inhibitorsstructure, transplacental transport, and safety studies in pregnancy.

Biologic Agent, Year of EMA Authorization, (Trade Name)	Structure	Transplacental Transport	Safety Studies
Infliximab, 1999, (Remicade, Remsima, Inflectra)	Chimeric human-murine complete IgG1 mAb	Active transport from gestational week 22 [23]	Bröms et al. 2016 [30] Casanova et al. 2013 [31] Shihab et al. 2016 [32] Weber-Schoendorfer [33]
Etanercept, 2000, (Enbrel, Benepali)	Fc fragment of human IgG1 fusion protein	Some [24]	Bröms et al. 2016 [30] Weber-Schoendorfer [33]
Adalimumab, 2003, (Humira)	Fully human complete IgG1 mAb	Active transport from gestational week 22 [23]	Burmester et al. 2017 [34] Bröms et al. 2016 [30] Casanova et al. 2013 [31] Shihab et al. 2016 [32] Weber-Schoendorfer [33]
Certolizumab, 2009, (Cimzia)	Humanized PEGylated Fab’ IgG fragment of mAb	Minimal [29]	Bröms et al. 2016 [30] Casanova et al. 2013 [31] Mariette et al. 2018 [29] Shihab et al. 2016 [32] Weber-Schoendorfer [33]
Golimumab, 2009, (Simponi)	Fully human complete IgG1 mAb	Active transport from gestational week 22 [35]	Bröms et al. 2016 [30] Weber-Schoendorfer [33]

EMA = European Medicines Agency; IgG = Immunoglobulin G; mAb = monoclonal antibody; Fab = antigen-binding fragment.

## Abbreviations

IgG	immunoglobulin G
MeSH	Medical Subject Headings
TNFR	Tumor Necrosis Factor receptor
Fc	Fragment crystallizable
IBD	Inflammatory Bowel Disease
TNFi	Tumor Necrosis Factor inhibitor
TNF	Tumor Necrosis Factor
Th-cells	T helper cells
Treg	T regulatory cells
IL	Interleukin
GRADE	Grading of Recommendations Assessment, Development and Evaluation
